# The energy and time saving coordinated control methods of CO_2_, VOCs, and PM_2.5_ in office buildings

**DOI:** 10.1371/journal.pone.0275157

**Published:** 2022-09-27

**Authors:** Xiaochun Wu, Yan Zhang, Fang Hou, Huichao Wang, Jianjie Zhou, Wei Yu

**Affiliations:** 1 School of Civil Engineering, Chongqing University, Chongqing, China; 2 Western Investment Limited Company of China Construction Third Engineering Bureau, Chengdu, Sichuan, China; 3 Joint International Research Laboratory of Green Buildings and Built Environments (Ministry of Education), Chongqing University, Chongqing, China; 4 National Centre for International Research of Low-carbon and Green Buildings (Ministry of Science and Technology), Chongqing University, Chongqing, China; Northeastern University (Shenyang China), CHINA

## Abstract

Indoor air pollution is complex and serious. In fact, an on-site investigation of an office building revealed that the concentration of three typical pollutants (CO_2_, VOCs, PM_2.5_) exceeded the Chinese standard. To identify a better control method to achieve good indoor air quality, an orthogonal experiment was carried out in an environmental chamber to compare the control time and energy consumption of four control methods (purifier+ and window+, purifier+ and window-, purified fresh air 240 m^3^/h and purified fresh air 400 m^3^/h) to meet the standard established for pollutants. The purifier+ and window+ method was found to be more effective in most conditions, with a control time reduced by 8.06% and energy consumption reduced by 11.91% compared with the traditional control method of purified fresh air 240 m^3^/h. This research highlights the optimal control strategy for the air quality in office buildings under different pollution conditions.

## 1 Introduction

Indoor air pollution is the third leading cause of air pollution worldwide, after bituminous coal and photochemical smog pollution [1]. High concentrations of indoor pollutants can lead to poor indoor air quality (IAQ), which has negative effects on human health and productivity [2,3]. Many investigations and studies have revealed an indoor CO_2_ level higher than 1000 ppm [4,5]. In residential buildings in Nanjing, China, the average indoor PM_2.5_ was 37 μg/m^3^ in summer and 56 μg/m^3^ in winter, with a significantly lower ratio of indoor and outdoor concentrations (I/O) in winter than in summer [6]. By reviewing indoor formaldehyde concentrations in China and other countries, Zhang [7] found that formaldehyde emission in Chinese cities, such as Beijing and Tianjin, far exceeded the limits set by the WHO [8]. Thus, indoor air pollution is quite serious in China. As workers spend more than eight hours per day in office buildings, creating a healthy and comfortable air environment is a priority.

The main sources of indoor pollutants are carbon dioxide (CO_2_), volatile organic compounds (VOCs), and fine particulate matter (PM_2.5_) [9]. CO_2_ is mainly derived from outdoor infiltration and people’s metabolism [10]. Artificial decoration materials used in the offices emit VOCs during the life cycle of the building [11]. Important sources of indoor PM_2.5_ include, but are not limited to, personnel activity and outdoor PM_2.5_ infiltration through the building envelope [12]. In office buildings, PM_2.5_ may also be generated from certain equipment, such as photocopiers, printers, computers, and other electronic equipment.

Three common strategies are currently used to improve the IAQ of buildings: air purifiers can significantly reduce the indoor concentration of particulate matter and VOCs [11,13–15]; natural ventilation is one of the most direct and effective strategies to remove and dilute indoor pollutants [16,17]; and fresh air systems are a complementary strategy to ensure indoor air quality. The ASHRAE Standard 62.2 suggests the use of mechanical ventilation systems to guarantee good building ventilation rate and indoor air quality [18]. Based on the above studies, natural ventilation, mechanical ventilation, and air purifiers are important control methods for reducing indoor pollutants.

The purification effectiveness of different control methods for a single pollutant has been extensively discussed. Li et al. [13] applied purifiers to reduce PM_2.5_ concentrations from different indoor sources. Based on their findings, the purification efficiency of air purifiers ranged from 43% to 86%, with indoor PM_2.5_ concentrations eventually stabilizing within 4 μg/m^3^. Turning on the air purifier significantly reduced indoor compound concentrations when the air change rate in the office building was low, with formaldehyde declining below the detection limit of 6.6 ppb [19]. HEPA filter-based air purifiers also evidently reduced aerosol concentration, even when operated for a short period (3 min) [20]. Intensified ventilation is an effective method for reducing CO_2_ concentrations in office buildings and improving indoor air quality [19,21]. According to Abdullah [16], when the window opening rate increased to 75%, the CO_2_ concentrations might be kept below 750 ppm during office working hours. When fresh air was continuously sent indoors, the CO_2_ concentration in the tested office was consistently below 700 ppm. As a result, Niu et al. [22] recommended the use of fresh air systems in office buildings for 4 h per day to ensure good indoor environment quality. Caron et al. [23] found that the average VOCs concentrations decreased by more than 80% when the room was converted from airtight to mechanically ventilated.

The purification effectiveness of control methods varies based on the pollutant for a complex situation of multiple pollutants. A field study [1] involving long-term monitoring of CO_2_, PM_2.5_, formaldehyde, and TVOC revealed that mechanical ventilation systems resulted in annual average concentrations of 640 ppm for CO_2_, 0.040 mg/m^3^ for formaldehyde, and 0.429 mg/m^3^ for TVOC in dwellings. These values are lower than those found in naturally ventilated residential buildings, except that of PM_2.5_, whose level was not as good as that obtained with the natural ventilation method. Quang et al. [24] found that a mechanical ventilation system with outdoor air filtration reduced indoor mean particle count and CO_2_ concentrations by 48% and 24%, respectively.

Previous studies compared the effect of single or coordinated control methods [1,13,25]. Indicators, such as air changes rate, post-control concentrations of pollutants, and energy consumption, are often used to evaluate the purification effectiveness of different control methods. Shi et al. [25] compared the energy consumption of PM_2.5_ purification using two control methods, air purifiers combined with open-window ventilation and fresh air units, and concluded that the fresh air units required a lower clean air delivery rate and consumed less electricity. Li et al. [13] compared the post-control concentrations of PM_2.5_ for four control methods, with concentrations ranked as natural attenuation > natural ventilation > air purifier > air purifier and natural ventilation. Huang et al. [1] compared control methods of medium- and high-efficiency filtration fresh air system and natural ventilation with air purifier in terms of the post-control concentration of PM_2.5_ and energy consumption. The model of natural ventilation with air purifier had a high performance, and could maintain an indoor PM_2.5_ concentration between 0.020 mg/m^3^ and 0.030 mg/m^3^ with lower energy consumption.

Overall, the purification effectiveness of various control methods for a single indoor air pollutant has been widely explored. However, previous studies mainly involved environmental chamber experiments for a single pollutant [26,27], with little comparison of the effectiveness and energy consumption of control methods for multiple pollutants (e.g., CO_2_, VOCs, and PM_2.5_) in the indoor environment. As a result, a uniform conclusion has not been formed for an integrated and effective control method for multiple pollutants. However, the purification effectiveness and energy consumption must be evaluated as the evaluation indicators of control methods. Multiple pollutants are complex and interactive, which pose a greater health risk to the human respiratory system [28]. Once their levels are exceeded, pollutants should be eliminated as quickly as possible. In addition, building energy consumption, especially for HVAC systems [29], accounts for a large amount of total carbon emissions [30]. A comparative study of the energy consumption of different control methods is thus helpful to identify the control method that consumes the least amount of energy to enable energy savings.

In response to the abovementioned problems, this study aimed to compare the purification effectiveness of coordinated control methods under typical multiple pollutants conditions and determine the optimal control methods with the shortest control time and lowest energy consumption. The coordinated control method involves opening windows, and turning on purifiers and purified fresh air. The findings of this study can fill the gap on coordinated control strategies for multiple pollutants, and provide guidance for future air environment management in office buildings.

This study opted to demonstrate the recommended control strategies for multiple pollutants through a combination of on-site investigation and Orthogonal experiment. Section 1 of this paper highlights the lack of theory on the coordinated control of multiple pollutants in existing studies. Section 2 elaborates on the experimental design and data analysis methods. Section 3 presents the results, which include the control time for single pollutants and multi-pollutants, energy consumption, comprehensive evaluation of the four control methods, and the recommended control methods. Section 4 further explains the reasons for the differences in the purification effect of the control methods and further improvement. Section 5 presents the study conclusion.

## 2 Methods

### 2.1 Experiment design

#### 2.1.1 On-site investigation

To determine the common control methods for indoor pollutants, an office building in Chongqing was selected to conduct the survey. The office building was built in 2015 and the investigation was conducted in 2019. A main road is located at the front of the building and good greenery is available within 50 m of the building; however, many car manufacturing plants are located in the district. The floor area of this office building is 120,000 square meters, which is the average area of general office buildings. The office building is equipped with typical functional rooms (single-person office, multi-person office, and conference room) and common air purification equipment (purified fresh air system and portable air purifiers). During the investigation, the monthly average temperature was 20–31°C and the humidity was 30–65% RH.

The participants were divided into four groups: single-person office users; multi-person office users; conference room users; and heating, ventilation, and air conditioning (HVAC) system operators. The first three groups of participants were questioned on their control of air purification equipment, their subjective evaluation of IAQ, and their behavioral habits to help improve IAQ. Details of the questionnaire are provided in S1 File. For the HVAC system operators, the operating time of the air conditioning system and air purification equipment in the building was evaluated.

To obtain the current pollution situation in this office building, the single-person office, multi-person office, and conference room on the sixth floor were selected, and the indoor and outdoor air qualities were continuously monitored for 2 weeks in the summer, autumn, and winter. The data obtained during the working hours of 8:00–17:00 were deemed valid. The parameters measured included temperature, humidity, and pollutants, such as CO_2_, VOCs, and PM_2.5_.

#### 2.1.2 Orthogonal experiment

The Orthogonal experiment was conducted in the environmental chamber of Chongqing University. The room size was follows: 6 m×5.3 m×2.8 m; windows: 2.85 m×1.1 m, 1 m from the ground; door: 0.95 m×2.1 m; door-side windows: 0.95 m×1.2 m, 0.9 m from the ground. During the experiment, only 1/4 window, whose area was 0.78 m^2^, was opened.

As the experiment was also carried out in the winter, the temperature of the air conditioner in the environment chamber was set to 18°C. The actual measured temperature was 18.4 ± 1.1°C and the humidity was 49.2 ± 1.8% RH. No experimenters were present in the environmental chamber during the experiment. Except the necessary instruments and equipment for the experiments, there was no furniture in the environmental chamber. The original concentration of pollutants in the environmental chamber was 400 ppm (CO_2_), less than 0.6 mg/m^3^ (VOCs), and less than 35 μg/m^3^ (PM_2.5_; both indoor and outdoor). Therefore, artificial pollution sources were created for CO_2_, VOCs, and PM_2.5_. Industrial CO_2_ with a purity of 99.5% was selected as the source of CO_2_ pollution. The outlet of the gas cylinder was placed after the head fan, mixed by the fan, and sent into the room. Florida Water was selected as the source of VOCs pollution. The amount of spray leads to an average VOCs concentration of up to 1–2 mg/m^3^ in the indoor breathing zone. The burning of a mosquito coil was used as the indoor and outdoor PM_2.5_ pollution source. Before the experiment, the experimenters would walk with the mosquito coil around the room to evenly distribute the indoor PM_2.5_. The mosquito coil was fixed behind the fan, and the fan’s reverse airflow was used to mix the air to form an even outdoor PM_2.5_ pollution source. It is assumed that no artificial source of pollutants existed in the room after the start of the experiment. As outdoor CO_2_ is relatively stable all year round, with 400–450 ppm, and outdoor VOCs has a low level of < 0.001 mg/m^3^, the condition of outdoor CO_2_ and VOCs exceeding the standard was not considered.

Before the initiation of the experiment, the windows and doors were closed, and the air conditioner, air purification equipment, and temperature, humidity, and IAQ sensors were switched on until the indoor concentrations of CO_2,_ VOCs, and PM_2.5_ were below the standard limits. Thereafter, the air purification equipment was switched off and the indoor air pollution sources were artificially created until the concentration of pollutants in the breathing zone exceeded the values set for each condition. After stabilization for 2 min, the air purification equipment was switched on again, and recording of the control time and energy consumption was initiated. When the monitoring data from all IAQ sensors showed that the indoor CO_2_ was below 600 ppm, the VOCs was below 0.6 mg/m^3^, and PM_2.5_ was below 35 μg/m^3^, the control time and energy consumption were re-recorded. After stabilization for 5 min, the experiment was completed for this condition. The detailed laboratory protocols are available at dx.doi.org/10.17504/protocols.io.3byl4bxpjvo5/v1.

**Fig 1:**
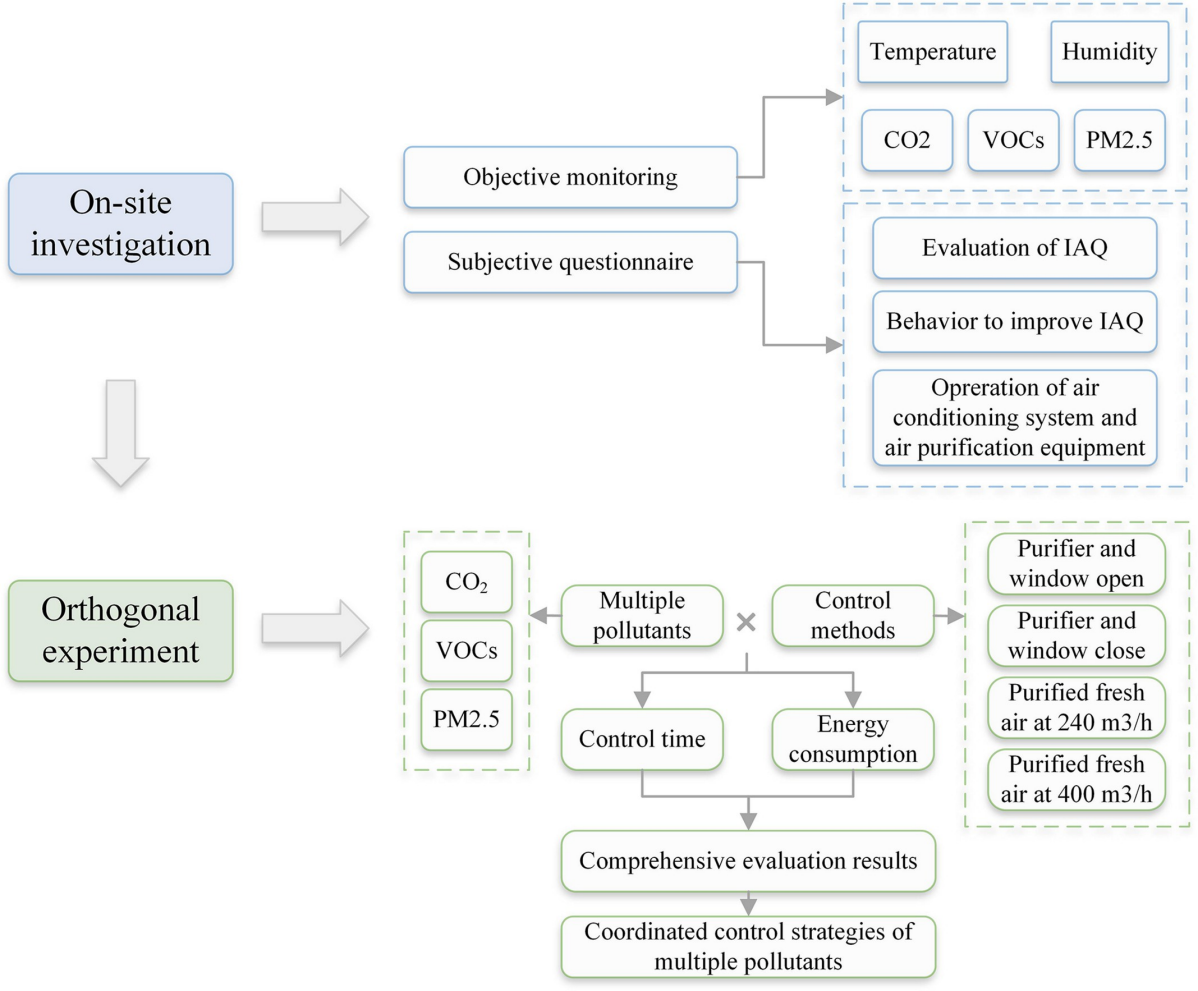
Flowchart of the methods section.

According to the Indoor Air Quality Standard (GB/T 18883–2002), the concentration of CO_2_ is limited to 1000 ppm, and that of VOCs is limited to 0.6 mg/m^3^ [31]. The indoor and outdoor PM_2.5_ pollution can be divided into three classes according to the Ambient Air Quality Standard (GB 3095–2012) [32]: 0–35 μg/m^3^ (excellent), 36–75 μg/m^3^ (good), and 76–115 μg/m^3^ (light pollution). According to the survey results, people tended to turn on mechanical fresh air, purifiers, or open the window when IAQ was not very good, which provides a reference for the establishment of the control method in the orthogonal experiment. Therefore, the purifier switched on and the window opened (purifier+ and window+), the purifier switched on and the window closed (purifier+ and window-), purified fresh air at 240 m^3^/h, and purified fresh air at 400 m^3^/h were set as the four typical control methods in this study. The five factors of CO_2_ concentration (two levels), indoor VOCs concentration (two levels), control methods (four levels), indoor PM_2.5_ concentration (three levels), and outdoor PM_2.5_ concentration (three levels) were orthogonalized. Finally, 16 operating conditions were employed, and the same thermal environment and pollution-free condition was established for comparison. The conditions of the orthogonal experiment are shown in Table 1. The flowchart of the on-site investigation and orthogonal experiment is shown in Fig 1.

**Table 1:** Orthogonal experiment condition and comprehensive evaluation result.

Condition	Experimental factors					Experimental results						
	CO_2_ (ppm)	VOCs (mg/m^3^)	Indoor PM_2.5_ (μg/m^3^)	Outdoor PM_2.5_ (μg/m^3^)	Control methods	Control time (min)	Energy consumption (kW‧h)	Percentage of time	Percentage of energy consumption	Comprehensive evaluation		
										PM_2.5_	CO_2_	VOCs
1	1000-(1)	0.6-(1)	0–35(1)	0–35(1)	Purified fresh air240(1)	contrast	0.059* ^a^	5.56%	8.13%	6.84%	7.36%	6.33%
2	1000-	0.6+(2)	0–35	0–35	purifier+ and window-(2)	50	0.257	27.78%	35.40%	31.59%	33.11%	30.07%
3	1000+(2)	0.6-	0–35	0–35	purifier+ and window+(3)	20	0.160	11.11%	22.04%	16.57%	18.76%	14.39%
4	1000+	0.6+	0–35	0–35	Purified fresh air400(4)	60	0.590	33.33%	81.27%	57.30%	66.89%	47.71%
5	1000-	0.6+	36–75(2)	0–35	purifier+ and window-	50	0.160	27.78%	22.04%	24.91%	23.76%	26.06%
6	1000+	0.6-	36–75	0–35	purifier+ and window+	20	0.187	11.11%	25.76%	18.43%	21.37%	15.51%
7	1000-	0.6-	76–115(3)	0–35	Purified fresh air240	13	0.031	7.22%	4.21%	5.72%	5.11%	6.32%
8	1000+	0.6+	76–115	0–35	Purified fresh air400	60	0.270	33.33%	37.19%	35.26%	36.03%	34.49%
9	1000-	0.6+	0–35	36–75(2)	purifier+ and window+	10	0.060	5.56%	8.21%	6.88%	7.42%	6.36%
10	1000+	0.6+	0–35	36–75	Purified fresh air240	60	0.584	33.33%	80.44%	56.89%	66.31%	47.46%
11	1000-	0.6-	36–75	36–75	Purified fresh air400	60	0.690	33.33%	95.04%	64.19%	76.53%	51.84%
12	1000+	0.6-	76–115	36–75	purifier+ and window-	180+	0.712+	100.00%	98.07%	99.04%	98.65%	99.42%
13	1000-	0.6-	0–35	76–115(3)	Purified fresh air400	0+	0.039*	5.56%	5.37%	5.46%	5.43%	5.50%
14	1000+	0.6-	0–35	76–115	purifier+ and window-	180+	0.726+	100.00%	100.00%	100.00%	100.00%	100.00%
15	1000+	0.6+	36–75	76–115	Purified fresh air240	60	0.271	33.33%	37.33%	35.33%	36.13%	34.53%
16	1000-	0.6+	76–115	76–115	purifier+ and window+	35	0.192	19.44%	26.45%	22.95%	24.35%	21.54%
17	1000-	0.6-	0–35	0–35	Close the window	contrast	0.251*	5.56%	34.57%	20.06%	25.87%	14.26%

### 2.2 Instrument measurements

The measurement instruments used in this experiment were calibrated. Temperature and humidity were recorded using the WSZY-1 temperature and humidity automatic recorder instrument. The respective temperature and humidity ranges were -40–100°C and 0–100% RH, and the accuracy was 0.1°C and 0.1% RH. The detection of CO_2_ and PM_2.5_ was carried out using the Brauntong BRT-Smart128s portable air quality detector. The PM_2.5_ range was 0–999 μg/m^3^, and its resolution was within 1 μg/m^3^. The CO_2_ range was 400–2000 ppm, and its resolution was within 3 ppm. The detection of VOCs was carried out using semiconductor sensors, with an accuracy of 0.001 mg/m^3^. Monitoring data were automatically recorded for 1 min in the on-site investigation and orthogonal experiment. The energy consumption of the air conditioner was measured using the micropower monitor, TECMAN, and the energy consumption of the air purification equipment was measured using Nortel T8. The accuracy of the two instruments was 0.01 W.

In the orthogonal experiment, a vertical cabinet air conditioner (model KFR-50LW/ (50569) Ba-3) was used to control the indoor thermal environment, and two purification devices were used to control the indoor air environment: cabinet-type full-heat purified fresh air equipment (model ZSH-Z-G00 (1705)) and humidified air purifier (model F-ZXJE90C). According to the size of the environmental chamber, the indoor personnel capacity was set to 8 persons. Therefore, the low-grade fresh air volume was set to 240 m^3^/h according to the Design Standard for HVAC in Civil Buildings (GB50736-2012) [33], and the high-grade was 400 m^3^/h. The clean air delivery rate of the purifier of the particles was 378 m^3^/h, and that of the VOCs was 208 m^3^/h.

**Fig 2:**
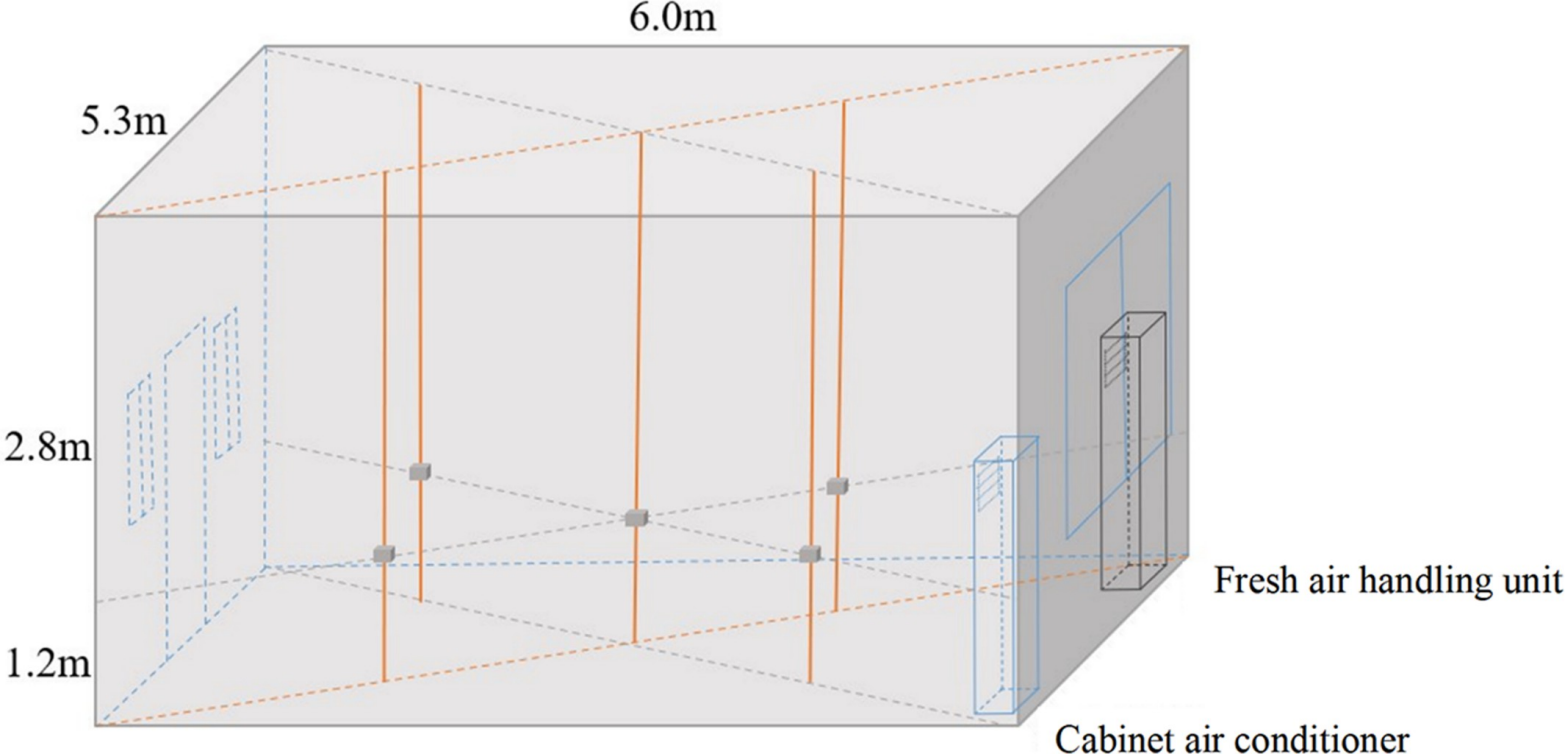
Experimental platform and monitoring points.

The position of the measuring points in the orthogonal experiment is shown in Fig 2. To monitor the indoor pollutants in the human breathing zone, a total of five measuring points were set at 1.2 m from the ground. Four measuring points were set at a distance of 1.0 m from the wall and one center point. Two measuring points were also set at the air inlet and outlet of the fresh air equipment, respectively.

### 2.3 Statistical analysis

The results of the orthogonal experiment were analyzed using the visual analysis method (S1 Table). The control time and energy consumption were combined to provide a comprehensive evaluation of the coordinated control effect of multiple pollutants. As the three types of pollutants have different impacts on personnel health, three types of comprehensive evaluations were performed, with the different indoor air pollutants as the control targets.

## 3 Results

### 3.1 Investigation results

Office workers were found to adopt a few measures to control the IAQ, and hardly turned on the air purification equipment. The single-person office was not equipped with air purifiers. Even if air purifiers are available in the multi-person office and conference room, people occasionally used these devices when the IAQ seemed really bad. Eighty percent of people were very concerned about the IAQ. During the winter, summer, and autumn, 100%, 80%, and 33% of people thought that the IAQ should be improved appropriately. Single-person office users preferred urgent improvement of the IAQ compared to multi-person office users. Although people had a strong willingness to improve IAQ, the opening of windows tended to be the control method employed.

The three available methods for the control of the indoor environment by officer users include natural ventilation, mechanical ventilation, and the use of air purifiers. Herein, the less common control method was the use of air purifiers with the opening of windows. The more common control methods were the opening of windows or use of an air purification equipment. The use of an air purifier with the closing of windows was also used in the office to ensure effective and quick reduction of the indoor concentration of VOCs and PM_2.5_.

The HVAC system in office building contains a multi-split air conditioner and independent fresh air equipment. The air conditioner was only used in summer and winter, and the fresh air equipment was only used in the transitional season. No fresh air existed indoors in the summer and winter. This air conditioning mode does not consider the IAQ.

The concentrations of CO_2_, VOCs, and PM_2.5_ are shown in Table 2. The three types of pollutants exceeded the Chinese standard at times. CO_2_ transiently exceeded the standard when people gathered in the conference room. VOCs exceeded the standard in single-person and multi-person offices. The concentration of PM_2.5_ in the multi-person office exceeded 35 μg/m^3^ in the three seasons, which might be due to the large number of persons and printers. For several abnormal situations, the high level of VOCs may be due to the perfumes and air fresheners used in the office, while the indoor PM_2.5_ pollution may be due to smoking.

**Table 2:** Indoor and outdoor concentration distribution of CO_2_, VOCs, and PM_2.5_ in office buildings.

Pollutants	Indoor pollutant concentration distribution		Concentration difference between indoor and outdoor
	Normal situation	Abnormal situation	
**CO**_**2**_	The CO_2_ is commonly 400–600 ppm. The CO_2_ during working hours (9:00–18:00) is higher than other periods.	Many high values appeared in the conference room which can reach 1500 ppm, and 2000 ppm once occurred.	The indoor CO_2_ is higher than the outdoor CO_2_.
**VOCs**	The VOCs in single-person office in summer exceeds the standard during non-working hours and significantly exceeds the standard throughout the day in winter.	The VOCs in single-person office in winter can reach up to 1.2 mg/m^3^. The VOCs in multi-person office in autumn are significantly higher than the standard between 14:00–16:00 and non-working hours, and its maximum can reach 0.8 mg/m^3^.	-^a^
**PM**_**2.5**_	The PM_2.5_ is commonly 20–90 μg/m^3^. The PM_2.5_ in multi-person office is higher than 35 μg/m^3^ in three seasons, and its concentration range in winter is 65–90 μg/m^3^. The PM_2.5_ in single-person office and conference room are sometimes higher than 35 μg/m^3^ in summer and winter. The indoor PM_2.5_ during working hours is higher than other periods.	Abnormal values mostly appear in multi-person office during working hours in summer and autumn, which can reach 100–300 μg/m^3^. The concentration of PM_2.5_ also exceeded 100 μg/m^3^ in single-person room in winter.	In summer and autumn, the outdoor PM_2.5_ is higher than indoors (the concentration difference is about 35 μg/m^3^), but the indoor PM_2.5_ in winter is higher than outdoors (the concentration difference is about 70 μg/m^3^).

^a^ Due to equipment placement problems, outdoor VOCs monitoring data is unreliable.

### 3.2 Control time required for a single pollutant

**Fig 3:**
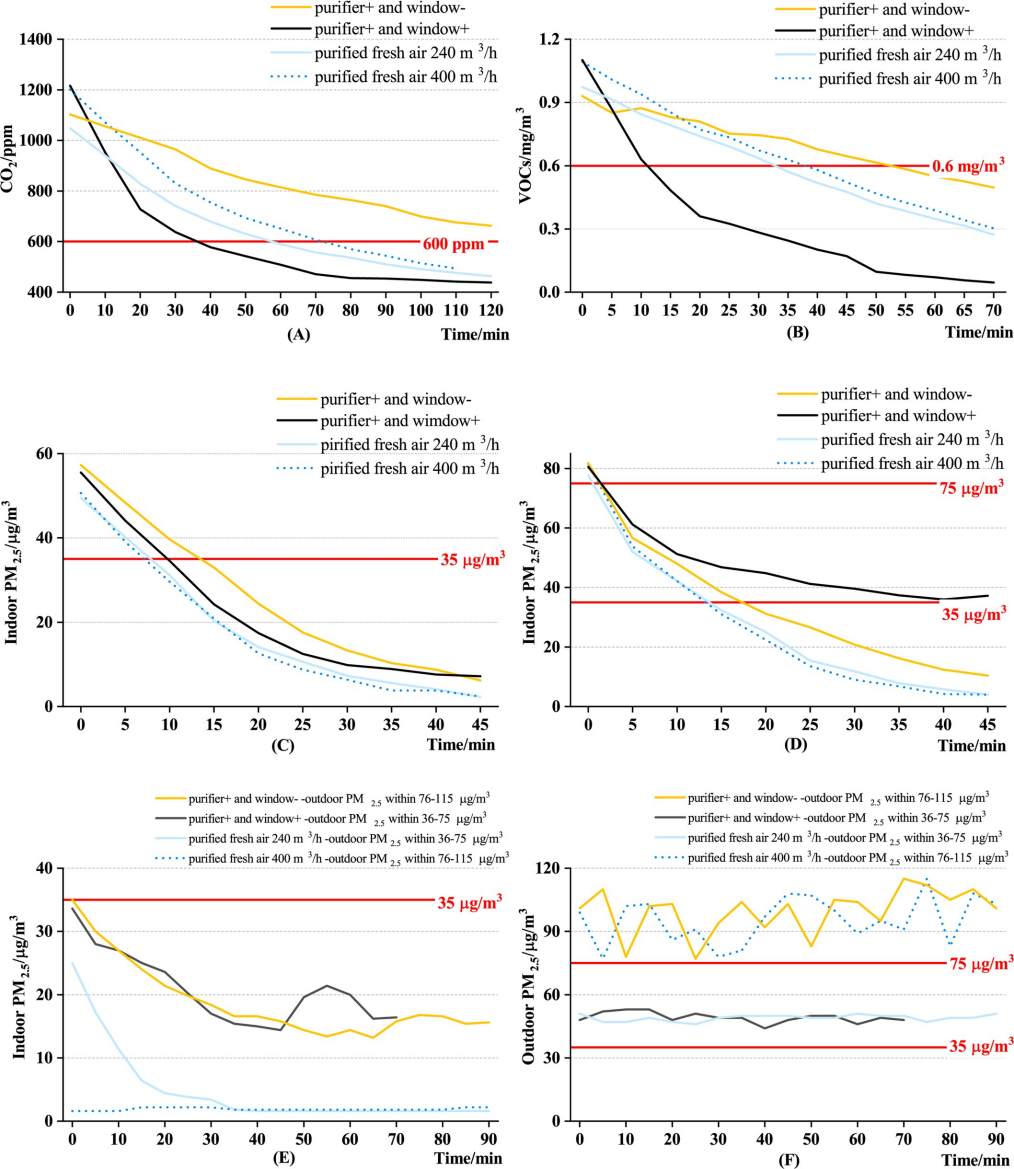
Comparison of the control methods under a single pollutant condition. (A) Indoor CO_2_ exceeding the standard. (B) Indoor VOCs exceeding the standard. (C) Indoor PM_2.5_ at a good level. (D) Indoor PM_2.5_ at a light pollution level. (E) Indoor PM_2.5_ concentration when outdoor PM_2.5_ exceeds the standard. (F) Outdoor PM_2.5_ concentration when outdoor PM_2.5_ exceeds the standard.

When the outdoor CO_2_ was 410–450 ppm and the indoor CO_2_ exceeded 1000 ppm, the concentration of CO_2_ was reduced using the four control methods, as shown in Fig 3A. In the first hour, the CO_2_ concentration decreased rapidly. When the CO_2_ was lower than 600 ppm, the decreasing rate became slow. The decrease in CO_2_ under purified fresh air 400 m^3^/h was faster than that under purified fresh air 240 m^3^/h. Under purifier+ and window+, the CO_2_ declined to 600 ppm within 0.5 h. However, in the same polluted environment, more than 1 h under purified fresh air and markedly more than 2 h under purifier+ and window- were required to reach a CO_2_ concentration of 600 ppm.

Fig 3B shows a comparison of the control effects of the four control methods when the VOCs exceed the standard. Under purifier+ and window+, VOCs declined from the initial concentration of 1 mg/m^3^ to 0.6 mg/m^3^ within 10 min, followed by the purified fresh air (30–35 min), and purifier+ and window- (50 min). Under purified fresh air, the indoor VOCs concentration decreased linearly with time, and the effect of increasing air volume on the rate of decrease of VOCs concentration was not apparent. The decreasing rate of indoor VOCs concentration under purified fresh air was 0.8, and was twice as high as that under the purifier+ and window-. The four control methods could reduce the VOCs concentration to less than 0.6 mg/m^3^ within 1 h. When the indoor concentration declined to 0.1–0.3 mg/m^3^ (outdoor VOCs concentration), the decreasing rate of VOCs slowed down.

When the indoor PM_2.5_ was 36–75 μg/m^3^, the four control methods reduce the PM_2.5_ concentration to a value below the standard within 15 min (Fig 3C); the control time of the purifier+ and window-, purifier+ and window+, and purified fresh air was 15 min, 10 min, and 8 min, respectively. The decreasing rate of the purifier+ and window+ was the largest, while that of the purifier+ and window- and purified fresh air was almost the same. The control time entirely depended on the initial PM_2.5_ concentration. When the indoor PM_2.5_ level was at light, the decreasing rate of PM_2.5_ was different owing to the purified fresh air volume (Fig 3D). When the purification air volume was 400 m^3^/h compared to 240 m^3^/h, the PM_2.5_ concentration significantly declined, with a good air quality level achieved within 5 min.

When the outdoor PM_2.5_ exceeded the standard (36–75 μg/m^3^ or 76–115 μg/m^3^) and the indoor PM_2.5_ was below the standard (0–35 μg/m^3^), purified fresh air could reduce the indoor PM_2.5_ from 25 μg/m^3^ to approximately 0 μg/m^3^ after 15 min (Fig 3E). The concentration of outdoor PM_2.5_ when the standard is exceeded is shown in Fig 3F. Outdoor lightly polluted air after purification and sent into the room will cause little increase in indoor PM_2.5_ concentration. Under purifier+ and window+, the control time was extended to 30 min, and the final indoor PM_2.5_ concentration could only be maintained at 15–20 μg/m^3^. Due to the PM_2.5_ pollution sources outdoors, the indoor PM_2.5_ will fluctuate when opening windows.

### 3.3 Control time required for multiple pollutants

When the IAQ was affected by binary pollutants, the difference in control time between various control methods was more distinct (Table 3). Purifier+ and window- could not control CO_2_ to meet the standard. When indoor VOCs and outdoor PM_2.5_ exist at the same time, the initial VOCs concentration determined the length of the control time. When the initial VOCs concentration increased from 0.7 mg/m^3^ to 1 mg/m^3^, the control time for purified fresh air at 240 m^3^/h extended from 5 min to 30 min, with a good level of outdoor PM_2.5_ maintained. During light indoor and outdoor PM_2.5_ pollution, although the purifier+ and window+ could reduce the indoor PM_2.5_ in the early stage, stabilization eventually occurred at 40 μg/m^3^, which will not result in a reduction of the indoor PM_2.5_ to a value below the standard.

**Table 3:** Comparison of control time required for binary pollutants.

Binary pollutants	Control method	Control time
**CO**_**2**_ **、VOCs**	purifier+ and window+	20 min
	Purified fresh air	40–60 min
	purifier+ and window-	-
**CO**_**2**_ **、indoor PM**_**2.5**_	purifier+ and window+	25 min
	Purified fresh air ^a^	60 min
**VOCs、indoor PM**_**2.5**_	purifier+ and window+	10 min
	Purified fresh air	35 min
	purifier+ and window-	50 min
**VOCs、outdoor PM**_**2.5**_	purifier+ and window+	25 min
	Purified fresh air 240	5 min/30 min
**indoorPM**_**2.5**_ **、outdoor PM**_**2.5**_	Purified fresh air	15 min
	purifier+ and window-	25 min
	purifier+ and window+	-

^a^ The absence of a number after the purified fresh air means that there is little difference in the control time between the two air volumes.

When the indoor environment was affected by four types of pollutants: CO_2_, VOCs, indoor PM_2.5_, and outdoor PM_2.5_, the control time of all conditions with CO_2_ pollution depended on the time required for CO_2_ reduction. Accordingly, the control time of the purifier+ and window+ was the shortest (approximately 1 h).

### 3.4 Energy consumption required for multiple pollutants

Conditions 1, 7, 9, and 13 had the lowest energy consumption (Table 1), within 0.06 kW‧h. Under these conditions, there were no air pollution sources indoors or only a single pollutant existed. Further, the purifier+ and window+ and purified fresh air 240 m^3^/h were the control methods, with lower energy consumption. Conditions 12 and 14 had the highest energy consumption when CO_2_ was the dominant pollutant, and the control method was purifier+ and window-. The energy consumption for these two conditions exceeded 0.7 kW‧h, which indicates that the purifier+ and window- may be an ineffective control method for CO_2_, with an excessively long control time, and thus a higher energy consumption. High energy consumption occurred in Conditions 4 and 10, approximately 0.6 kW‧h, when CO_2_ remained as the dominant pollutant. As the control method was purified fresh air, purified fresh air is insufficient for CO_2_ reduction. The concentration of indoor pollutants is an independent variable of energy consumption in the control process. The higher the pollution concentration and the more complex the combination of pollutants, the higher the energy consumption. However, the lower the number and lower the equipment power consumed in the control method, the lower the energy consumption. On the principle of the same IAQ control requirements, the energy consumption per 10 min required for the four control methods was ranked as follows from high to low (Table 4): purifier+ and window->purified fresh air 400 m^3^/h>purified fresh air 240 m^3^/h>purifier+ and window+.

**Table 4:** Energy consumption levels required by the four control methods under the same IAQ control requirements.

Control method	Energy consumption kW·h/10 min				Average energy consumption kW·h/10 min
	CO_2_	VOCs	Indoor PM_2.5_	Outdoor PM_2.5_	
**purifier+ and window+**	0.029	0.021	0.032	0.021	0.026
**Purified fresh air 240**	0.071	0.071	0.025	0.071	0.060
**Purified fresh air 400**	0.072	0.072	0.080	0.061	0.071
**purifier+ and window-**	0.120	0.035	0.073	0.120	0.087

### 3.5 Comprehensive evaluation results

Based on the control objectives for the three pollutants, a comprehensive evaluation of different control methods was performed. The less the control time and the more energy-saving the control method, the smaller the comprehensive evaluation index value, which indicates a better control method.

1. For CO_2_ exceeding the standard, as CO_2_ is harmless to the human body, the primary consideration factor is energy consumption. Table 1 shows that Condition 3 has the minimum comprehensive evaluation value, while Condition 14 has the highest comprehensive evaluation value. The control method ranking from good to bad was purifier+ and window+ → Purified fresh air 400 m^3^/h → Purified fresh air 240 m^3^/h → purifier+ and window-. The control time of the purifier+ and window+ was 20 min, and its energy consumption was 0.16 KW·h.
2. For VOCs concentration exceeding the standard, as VOCs are harmful to the human body, the primary consideration factor is control time. Condition 9 had the minimum comprehensive evaluation value, while Condition 4 had the highest comprehensive evaluation value. The control method ranking from good to bad was purifier+ and window+ → purifier+ and window- → Purified fresh air 400 m^3^/h → Purified fresh air 240 m^3^/h. The control time of the purifier+ and window+ was 10 min, and its energy consumption was 0.0596 KW**·**h.
3. For PM_2.5_ pollution indoors, Condition 7 had the minimum value of the comprehensive evaluation, indicating that purified fresh air 240 m^3^/h is the best control method, with a control time of 13 min, and energy consumption of 0.0306 KW**·**h. For PM_2.5_ pollution outdoors, the comprehensive evaluation value of Condition 13 was the smallest, indicating that purified fresh air 400 m^3^/h is the best control method, with a control time of 10 min, and energy consumption of 0.039 KW**·**h. Of note, only a single pollutant was found in Conditions 7 and 13, leading to a better effect of purified fresh air than the purifier+ and window+. For PM_2.5_ pollution indoors and outdoors, the comprehensive evaluation value of Condition 16 was the smallest when the control method was purifier+ and window+, with the control time extending to 35 min, and the energy consumption increasing to 0.192 KW**·**h. Therefore, with PM_2.5_ pollution, the effectiveness of the control method depends on the severity of the air pollution.

According to the on-site investigation, purified fresh air equipment is commonly used during the transition season. Therefore, the purified fresh air 240 m^3^/h is regarded as the traditional control method, and the purifier+ and window+ is regarded as the optimal control method for comparison. When CO_2_ pollution occurred indoors, the purifier+ and window+ could reduce the control time by 40 min (22.22%) and save electricity by 0.254 KW**·**h (34.99%) compared with the traditional control method. When VOCs pollution occurred indoors, the purifier+ and window+ reduced the control time by 37.5 min (20.83%) and saved 0.3017 KW**·**h of electricity (41.56%). When indoor and outdoor PM_2.5_ pollution simultaneously occurred, the purifier+ and window+ was 25 min faster than the traditional method (13.89%), and the amount of saved energy consumption was 0.079 KW**·**h (10.88%). In general, considering various air pollution conditions, the control time of purifier+ and window+ was 12 min (8.06%) shorter than that of purified fresh air 240 m^3^/h, and the amount of energy consumption saved was 0.0865 KW**·**h (11.91%).

In summary, through a comprehensive evaluation, the best control method was identified as purifier+ and window+, with less control time and less energy consumption in most cases, followed by purified fresh air 240 m^3^/h. Compared to the control method of increasing the fresh air volume to 400 m^3^/h, little difference in the control time was found between the two; however, the energy consumption was twice that of the low air volume. The purifier+ and window- method was identified as the worst method.

### 3.6 Recommended control strategies for multiple pollutants

Based on the comprehensive evaluation results, the recommended control methods are proposed for different air pollution conditions (Table 5). As long as the outdoor PM_2.5_ reaches an excellent level, purifier+ and window+ is recommended to remove other pollutants in the room, especially CO_2_. The purifier+ and window+ or purified fresh air 240 m^3^/h is recommended for indoor VOCs pollution. When the outdoor air quality is excellent, the use of fresh air alone is feasible. At this time, the control time extends to approximately 35 min; however, the lowest energy consumption is achieved with this method. For indoor PM_2.5_ pollution, the recommended control method is purifier+ and window+ or fresh air without filtration. When the outdoor PM_2.5_ reaches light pollution level, purifier+ and window- or purified fresh air should be selected, with purifier+ and window- requiring less energy consumption. At this time, opening of windows is not recommended. For both indoor and outdoor PM_2.5_ pollution, the purifier+ and window- and purified fresh air 240 m^3^/h are more effective. The control time of the purified fresh air depends on the severity of air pollution.

**Table 5:** Recommended control methods.

Condition	CO_2_	VOCs	Indoor PM_2.5_	Outdoor PM_2.5_	Recommended control methods	Minimum control time	Control methods for optimal energy consumption
**1**	1000-	0.6-	0–35	0–35	open the window	-	-
**2**	1000+	0.6-	0–35	0–35	purifier+ and window+	20 min	
**3**	1000-	0.6+/ 1.0	0–35	0–35	purifier+ and window+	10 min	
**4**	1000-	0.6-	36-75/ 50	0–35	purifier+ and window+/fresh air 240	8 min	purified fresh air ^a^
**5**	1000-	0.6-	76-115/80	0–35	fresh air 240	15 min	
**6**	1000-	0.6-	0–35	36-75/ 50	purified fresh air /Purifier	-	purifier+ and window-
**7**	1000-	0.6-	0–35	76-115/ 80	purified fresh air /purifier+ and window-	-	purifier+ and window-
**8**	1000+	0.6+	0–35	0–35	purifier+ and window+	20 min	
**9**	1000+	0.6-	36–115	0–35	purifier+ and window+	25 min	
**10**	1000-	0.6+	36–75	0–35	purifier+ and window+/fresh air	10 min/ 35 min	fresh air ^b^
**11**	1000-	0.6+	76–115	0–35	purifier+ and window+/fresh air	15 min/ 35 min	fresh air
**12**	1000-	0.6+	0–35	36–115	Purified fresh air 240	35 min	
**13**	1000-	0.6-	36–75	36–115	purifier+ and window- /Purified fresh air 240	25 min/ 15 min	purified fresh air 240
**14**	1000-	0.6-	76–115	36–115	purifier+ and window-/ Purified fresh air 240	25 min/ 30 min	purifier+ and window-
**15**	1000+	0.6+	36–115	0–35	purifier+ and window+	30 min	
**16**	1000+	0.6+	0–35	36–75	purifier+ and window+	30 min	
**17**	1000+	0.6+	36–75	76–115	purified fresh air 240	60 min	

^a^ "Purified fresh air" means that the ventilation volume is not less than the rated fresh air volume in the HVAC system design standard.

^b^ "Fresh air" means that the fresh air is induced without high efficiency filtration.

## 4 Discussion

### 4.1 Status of indoor air pollution

In this study, three typical pollutants in office buildings were monitored. The concentration of CO_2_ was found to range from 400–600 ppm; that of VOCs ranged from 0–0.8 mg/m^3^; and that of PM_2.5_ ranged from 20–90 μg/m^3^, except for abnormal conditions. The indoor air pollution of six office buildings in Chengdu, China [34] was found to be very close to our results, with the mean PM_2.5_ value ranging from 35–97 μg/m^3^ and mean CO_2_ value ranging from 462–572 ppm. This finding may be due to the two cities being geographically close, and possessing similar climatic environments. The indoor CO_2_ concentration in this on-site investigation was lower than that in Delhi, India [35]. However, the particulate matter concentration was markedly higher than that in Australia (14–42 μg/m^3^) [36] and Europe (2.7–32 μg/m^3^) [37], suggesting the need to improve the IAQ of office buildings in China. Miao et al.[38] found that indoor CO_2_ and TVOC pollution in four Chinese cities was not markedly severe; however, the PM_2.5_ and PM_10_ concentrations exceeded Chinese standards. This study also found that PM_2.5_ pollution was common in office buildings in the winter, especially multi-person offices, due to the reduced intake of fresh air when the HVAC system is turned on.

### 4.2 Reasons for the different effects of the control methods

According to the comprehensive evaluation result, the purifier+ and window+ is the optimal control method. Numerous studies have demonstrated the effectiveness of air purifiers in removing indoor particulate matter [39,40] and VOCs [41,42]. The indoor PM_2.5_ is significantly different between two spaces where the purifier is used or not used [14]. Using air purifiers equipped with high-efficiency particulate air filters can ensure purification, despite the presence of PM_2.5_ pollutants indoors and outdoors [43]. According to Gaur et al. [44], purifiers and ventilation led to a significant reduction in TVOC concentrations. Kolarik et al. [19] verified that the use of air purifiers in the presence of pollutant emissions from building materials and furniture could improve the IAQ through subjective and objective tests. The IAQ is perceived to be fresher and more acceptable when the air purifier is switched on than off. The above studies indicate that using purifiers is an effective strategy to control indoor pollutants.

Moderate ventilation is an effective method to control indoor pollutants. Indoor pollutant concentrations usually decrease when the air exchange rate increases [45], unless the I/O is > 1. Danish researchers reported that ventilation levels are too low in institutional buildings, resulting in CO_2_ exceeding the limit of 1000 ppm [5]. This study verified that the use of purifiers coupled with moderate ventilation has a better control effect. However, this control method will fail in the presence of outdoor PM_2.5_ pollution as the opening of windows will cause outdoor PM_2.5_ to constantly enter the room. The indoor PM_2.5_ can only become stable and slightly lower than the outdoor PM_2.5_ when the increasing rate of indoor PM_2.5_ and the purification rate of the purifier reach a dynamic balance. At this point, the effectiveness of indoor air pollution control depends on the "cleaning load" of the purifier. During episodic outdoor air pollution, ventilation can seriously affect the IAQ, and the higher the rate of air exchanges, the more serious the indoor air pollution [17].

Among the four control methods, the purifier+ and window- is the most ineffective, especially when the CO_2_ exceeds the standard. The closing of windows allows CO_2_ to transfer only through the gaps in the envelope, weakening the diffusion driven by the concentration difference. The rate of air exchange of purifier+ and window- is 4.3 h^-1^, which is less than the purified fresh air (4.5 h^-1^) and even less than the purifier+ and window+ (4.8 h^-1^). Therefore, the CO_2_ removal efficiency of the purifier+ and window- is the lowest.

In summary, the effective control method depends on the indoor and outdoor pollution sources. When CO_2_, VOCs, or PM_2.5_ pollution exists indoors and the I/O ratio is <1, the purifier+ and window+ method is recommended. When outdoor PM_2.5_ pollution is severe, using a purifier or purified fresh air and simultaneously closing windows can combine the advantages of short control time and low energy consumption.

### 4.3 Applications

This study revealed the efficient and low-energy control methods for different multi-pollutant conditions, providing a reference for designers, managers, and researchers.

First, designers should reserve the space for installing purified fresh air systems in the design of air conditioning systems for office buildings; this is because the preferable control method is purified fresh air 240 m^3^/h in the presence of outdoor PM_2.5_ pollution.

Second, this study proposes recommended control methods with the shortest control time and least energy consumption for multiple pollutants, which can guide office building managers in the prompt switching on or off of the purified fresh air system according to the indoor air environment monitoring data.

Third, these findings can be generalized to similar situations on the indoor air environment in office buildings. However, before application, the environment and intervention consistency must be considered [46]. Environment consistency means that the application scenario is an office building of similar geographical location, room functions, and building volumes. Intervention consistency means that the application scenario should be available to purifiers and purified fresh air systems, and these facilities should be used effectively over time. If these factors are not consistent with those of this study, multiple air pollutants need to be detected, and the control methods must be modified according to the specific application scenarios.

### 4.4 Limitations

This study had some limitations. As the air conditioner used in the orthogonal experiment runs intermittently, indoor and outdoor air exchange is strengthened when the air conditioner is being used. In contrast, the indoor airflow disturbance is weakened when the air conditioner is on standby. Therefore, the concentration of indoor PM_2.5_ fluctuates significantly, which may bias the control time and energy consumption. Other indoor air pollutants, such as CO, NOx, and O_3_, were not monitored in this study. Atmospheric oxidation of VOCs in the presence of NOx leads to the formation of secondary organic aerosols in PM_2.5_ [47,48]. Therefore, indoor air pollution should be comprehensively evaluated in future studies and the interaction between different pollutants should be clarified. In this study, the orthogonal experiment was only conducted in the winter. Thus, the seasonal effect needs to be considered in the future. Through the on-site investigation, indoor VOCs and PM_2.5_ pollution levels were more severe in winter and all participants believed that the IAQ should be improved in winter. Thus, conducting orthogonal experiments in winter is a priority and urgent need.

## 5 Conclusions

The indoor pollutants in office buildings are complex and difficult to effectively control. In this study, CO_2_, VOCs, and PM_2.5_ were employed as the target pollutants and the optimal coordinated control methods were discussed under the conditions of multiple pollutants. The main conclusions are as follows:

When multiple pollutants coexist and CO_2_ is the main removal target, the recommended control method is purifier+ and window+, with a control time of 20–30 min and energy consumption within 0.19 kW·h.

When multiple pollutants coexist and VOCs are the main removal targets, the recommended control method is purifier+ and window+, with a control time of 10–30 min and energy consumption within 0.20 kW·h. Alternatively, fresh air can be used for the least amount of energy consumption.

When multiple pollutants coexist and PM_2.5_ is the main removal target, the recommended control method is based on the existence of outdoor PM_2.5_ pollution. If there is no outdoor PM_2.5_ pollution, the recommended control method is purifier+ and window+, with a control time of 10–30 min and energy consumption within 0.19 kW·h. Alternatively, fresh air can be used for the least energy consumption. If outdoor PM_2.5_ pollution exists, windows should not be opened, and the control method of purifier+ and window- or purified fresh air 240 m^3^/h is recommended, with the shortest control time of 25 min for purifier+ and window- and 15 min for purified fresh air 240 m^3^/h.

Generally, during the coexistence of CO_2_, VOCs, and PM_2.5_ pollution, the purifier+ and window+ method is better than the other common control methods (purifier+ and window-, purified fresh air 240 m^3^/h, and purified fresh air 400 m^3^/h). Compared with the traditional control method, the control time of purifier+ and window+ is reduced by 8.06% and the amount of energy consumption saved is 11.91%.

Through a comprehensive evaluation, this study revealed the recommended control methods for the coexistence of multiple pollutants. The increased use of portable air purifiers is suggested to improve the purification effectiveness of multiple pollutants in future buildings. Our results will guide the design and application of air purification systems in office buildings and encourage office workers to use the air purification equipment. Overall, this study provides guidance for designers, managers, and researchers on the comprehensive management of indoor multi-pollutants, which is significant for creating a good indoor air environment and ensuring good health.

## Supporting information

S1 Table Orthogonal experiment condition and comprehensive evaluation result table. (DOCX)

S1 File Questionnaire administered to people in the office building. (DOCX)

## Abbreviations

CO_2_: Carbon Dioxide
VOCs: Volatile Organic Compounds
PM_2.5_: Particulate Matter, where particles have an aerodynamic diameter equal to or less than 2.5 μm
IAQ: Indoor Air Quality
I/O: Ratio of Indoor and Outdoor Concentrations
HVAC: Heating, Ventilation, and Air Conditioning
