# Supplementary material for: The energy and time saving coordinated control methods of CO2, VOCs, and PM2.5 in office buildings
Source: PLoS One. 2022 Sep 27;17(9):e0275157. doi: 10.1371/journal.pone.0275157 (PMC9514625; doi:10.1371/journal.pone.0275157)
Supplement: S1 Table — (DOCX) [file pone.0275157.s001.docx]

**S1 Table. Orthogonal experiment condition and comprehensive evaluation result table**

| Condition | | Experimental factors | | | | | Experimental results | | | | | | |
| --- | --- | --- | --- | --- | --- | --- | --- | --- | --- | --- | --- | --- | --- |
|  |  | CO_2_ | Indoor PM_2.5_ | Outdoor PM_2.5_ | VOCs | Control methods | Control time | Energy consumption | Percentage of time | Percentage of energy consumption | Comprehensive evaluation | | |
|  |  |  |  |  |  |  |  |  |  |  | PM_2.5_ | CO_2_ | VOCs |
| 1 | | 1000-（1） | 0-35（1） | 0-35（1） | 0.6-（1） | Purified fresh air240（1） | contrast | 0.059* ^a^ | 5.56% | 8.13% | 6.84% | 7.36% | 6.33% |
| 2 | | 1000- | 0-35 | 0-35 | 0.6+（2） | Purifier+ and window-（2） | 50 | 0.257 | 27.78% | 35.40% | 31.59% | 33.11% | 30.07% |
| 3 | | 1000+（2） | 0-35 | 0-35 | 0.6- | Purifier+ and window+（3） | 20 | 0.160 | 11.11% | 22.04% | 16.57% | 18.76% | 14.39% |
| 4 | | 1000+ | 0-35 | 0-35 | 0.6+ | Purified fresh air400（4） | 60 | 0.590 | 33.33% | 81.27% | 57.30% | 66.89% | 47.71% |
| 5 | | 1000- | 36-75（2） | 0-35 | 0.6+ | Purifier+ and window- | 50 | 0.160 | 27.78% | 22.04% | 24.91% | 23.76% | 26.06% |
| 6 | | 1000+ | 36-75 | 0-35 | 0.6- | Purifier+ and window+ | 20 | 0.187 | 11.11% | 25.76% | 18.43% | 21.37% | 15.51% |
| 7 | | 1000- | 76-115（3） | 0-35 | 0.6- | Purified fresh air240 | 13 | 0.031 | 7.22% | 4.21% | 5.72% | 5.11% | 6.32% |
| 8 | | 1000+ | 76-115 | 0-35 | 0.6+ | Purified fresh air400 | 60 | 0.270 | 33.33% | 37.19% | 35.26% | 36.03% | 34.49% |
| 9 | | 1000- | 0-35 | 36-75（2） | 0.6+ | Purifier+ and window+ | 10 | 0.060 | 5.56% | 8.21% | 6.88% | 7.42% | 6.36% |
| 10 | | 1000+ | 0-35 | 36-75 | 0.6+ | Purified fresh air240 | 60 | 0.584 | 33.33% | 80.44% | 56.89% | 66.31% | 47.46% |
| 11 | | 1000- | 36-75 | 36-75 | 0.6- | Purified fresh air400 | 60 | 0.690 | 33.33% | 95.04% | 64.19% | 76.53% | 51.84% |
| 12 | | 1000+ | 76-115 | 36-75 | 0.6- | Purifier+ and window- | 180+ | 0.712+ | 100.00% | 98.07% | 99.04% | 98.65% | 99.42% |
| 13 | | 1000- | 0-35 | 76-115（3） | 0.6- | Purified fresh air400 | 0+ | 0.039* | 5.56% | 5.37% | 5.46% | 5.43% | 5.50% |
| 14 | | 1000+ | 0-35 | 76-115 | 0.6- | Purifier+ and window- | 180+ | 0.726+ | 100.00% | 100.00% | 100.00% | 100.00% | 100.00% |
| 15 | | 1000+ | 36-75 | 76-115 | 0.6+ | Purified fresh air240 | 60 | 0.271 | 33.33% | 37.33% | 35.33% | 36.13% | 34.53% |
| 16 | | 1000- | 76-115 | 76-115 | 0.6+ | Purifier+ and window+ | 35 | 0.192 | 19.44% | 26.45% | 22.95% | 24.35% | 21.54% |
| 17 | | 1000- | 0-35 | 0-35 | 0.6- | Close the window | contrast | 0.251* | 5.56% | 34.57% | 20.06% | 25.87% | 14.26% |
| Time | K1 | 27.75 | 47.5 | 34.13 | 59.13 | 33.25 |  |  |  |  |  |  |  |
|  | K2 | 80 | 47.5 | 77.5 | 48.13 | 115 |  |  |  |  |  |  |  |
|  | K3 |  | 72 | 68.75 |  | 21.25 |  |  |  |  |  |  |  |
|  | K4 |  |  |  |  | 45 |  |  |  |  |  |  |  |
|  | Range | 52.25 | 24.5 | 43.37 | 11 | 93.75 |  |  |  |  |  |  |  |
| Energy consumption | K1 | 0.186 | 0.309 | 0.214 | 0.325 | 0.236 |  |  |  |  |  |  |  |
|  | K2 | 0.438 | 0.327 | 0.511 | 0.298 | 0.464 |  |  |  |  |  |  |  |
|  | K3 |  | 0.301 | 0.307 |  | 0.150 |  |  |  |  |  |  |  |
|  | K4 |  |  |  |  | 0.397 |  |  |  |  |  |  |  |
|  | Range | 0.252 | 0.026 | 0.297 | 0.027 | 0.314 |  |  |  |  |  |  |  |
| Comprehensive evaluation (PM2.5) | K1 | 21.07% | 35.19% | 24.58% | 39.53% | 26.19% |  |  |  |  |  |  |  |
|  | K2 | 52.35% | 35.72% | 56.75% | 33.89% | 63.88% |  |  |  |  |  |  |  |
|  | K3 |  | 40.74% | 40.93% |  | 16.21% |  |  |  |  |  |  |  |
|  | K4 |  |  |  |  | 40.55% |  |  |  |  |  |  |  |
|  | Range | 31.28% | 5.55% | 32.17% | 5.64% | 47.67% |  |  |  |  |  |  |  |
| Comprehensive evaluation (CO2) | K1 | 22.88% | 38.16% | 26.55% | 41.65% | 28.73% |  |  |  |  |  |  |  |
|  | K2 | 55.52% | 39.45% | 62.23% | 36.75% | 63.88% |  |  |  |  |  |  |  |
|  | K3 |  | 41.04% | 41.48% |  | 17.98% |  |  |  |  |  |  |  |
|  | K4 |  |  |  |  | 46.22% |  |  |  |  |  |  |  |
|  | Range | 32.64% | 2.88% | 35.68% | 4.90% | 45.90% |  |  |  |  |  |  |  |
| Comprehensive evaluation (VOCs) | K1 | 19.25% | 32.23% | 22.61% | 37.41% | 23.66% |  |  |  |  |  |  |  |
|  | K2 | 49.19% | 31.99% | 51.27% | 31.03% | 63.88% |  |  |  |  |  |  |  |
|  | K3 |  | 40.44% | 40.39% |  | 14.45% |  |  |  |  |  |  |  |
|  | K4 |  |  |  |  | 34.89% |  |  |  |  |  |  |  |
|  | Range | 29.94% | 8.45% | 28.66% | 6.38% | 49.43% |  |  |  |  |  |  |  |

^a^ The conditions marked with "*" (Condition 1, Condition 13, and Condition 17) means that the energy consumption is calculated by running for 10 minutes since the indoor pollutants does not exceed the standard.
