# Supplementary material for: The energy and time saving coordinated control methods of CO2, VOCs, and PM2.5 in office buildings
Source: PLoS One. 2022 Sep 27;17(9):e0275157. doi: 10.1371/journal.pone.0275157 (PMC9514625; doi:10.1371/journal.pone.0275157)
Supplement: S1 File — (DOCX) [file pone.0275157.s002.docx]

**S1 File. Questionnaire administered to people in the office building**

Thank you for completing this questionnaire!

The following questions are designed to investigate your behaviour in this room.

| Room Date Number of people inside Weather outside |
| --- |
| A. When you are indoors |
| B. When you feel the indoor air quality is not good |
| C. When there are a lot of people indoors |
| D. Occasionally, no specific circumstances |
| E. No air purification equipment indoors |
| F. Other: |
| 1. Are you concerned about indoor air quality？(single choice) |
| A. Very concerned |
| B. Generally |
| C. Not concerned |
| 1. What are your expectations of the current indoor environment? (single choice) |
| A. Very good, no need to improve |
| B. Need to improve properly |
| C. Need to improve urgently |
| 1. When do you think the indoor air quality of the office room is not so good? (single choice) |
| A. When enter the office in the morning |
| B. On the sunny day after the rain |
| 4a. At this time, you are used to: (multiple choices) |
| A. Opening the windows |
| B. Turning on the air conditioner |
| C. Turning on the air purification equipment |
| 1. When do you think the indoor air quality of the meeting room is not so good? (single choice) |
| A. When enter the office in the morning |
| B. On the sunny day after the rain |
| 5a. At this time, you are used to: (multiple choices) |
| A. Opening the windows |
| B. Turning on the air conditioner |
| C. Turning on the air purification equipment |
